# Supplementary material for: Detection and Molecular Characterization of Enteric Viruses in Poultry Flocks in Hebei Province, China
Source: Animals (Basel). 2022 Oct 21;12(20):2873. doi: 10.3390/ani12202873 (PMC9598388; doi:10.3390/ani12202873)
Supplement: Supplementary file 1 [file animals-12-02873-s001.zip › animals-1959292-supplementary.pdf]

animals

Article

Detection and Molecular Characterization of Enteric Viruses in Poultry Flocks in Hebei Province, China

Libao Chen [1], Ligong Chen [1], Xuejing Wang [2,*], Shuying Huo [1] and Yurong Li [1,*]

[1] College of Veterinary Medicine, Hebei Agricultural University, Veterinary Biological Technology Innovation Center of Hebei Province, Baoding 071001, China
[2] Institute of Animal Husbandry and Veterinary Medicine of Hebei Province, Baoding 071000, China
* Correspondence: wangxuejing771226@163.com (X.W.); liyurong0312@126.com (Y.L.); Tel.: +86-13730474185 (X.W.); +86-15930207103 (Y.L.)

Supplementary Table S1: The Information of 212 samples (including 145 positive and 67 negative samples).

No.	Place/Year	age	Code Name	ChPV	ANV	CAstV	FAdV-I	ARoV	IBV	ARe	Viruses	Clinical signs
	The information of broiler (2019-2020)											
b1	Baoding/ 2019/BC	05w34d	C3	+			+				2	DP
b2	Baoding/ 2020/BC	04w28d	CLB129	+	+						2	St
b3	Baoding /2020/BC	04w25d	CLB88	+							1	DP
b4	Baoding/2020/BC	04w28d	CLB123	+							1	RP
b5	Baoding /2019/BC	05w30d	CLB37	+							1	DP
b6	Baoding/2019/BC	03w15d	CLB82	+	+						2	NR
b7	Baoding/2019/BC	04w27d	CLB29	+							1	St
b8	Baoding /2020/BC	05w33d	CLB6		+	+					2	NR
b9	Baoding/2019/BC	03w15d	CLB36	+	+						2	St
b10	Baoding/2019/BC	05w35d	C7	+		+					2	DP RP
b11	Baoding/2020/BC	05w35d	CLB64	+							1	DP
b12	Baoding/2020/BC	03w15d	CLB77	+	+						2	DP
b13	Baoding/2020/BC	05w34d	CLB70	+							1	DP
b14	Baoding/2019/BC	04w26d	CLB32	+	+						2	St RP
b15	Hengshui/2019/BC	04w28d	CLB96	+		+					2	St
b16	Hengshui/2020/BC	04w27d	CLB120	+	+						2	St
b17	Shijiazhuang/2020/BC	04w27d	CLB111		+						1	RP
b18	Shijiazhuang/2019/BC	04w24d	CLB51	+	+						2	St DP
b19	Shijiazhuang/2019/BC	05w32d	CLB15	+							1	DP
b20	Zhangjiakou/2019/BC	05w32d	CLB18			+					1	DP
b21	Zhangjiakou/2019/BC	05w34d	CLB17	+	+	+					3	DP
b22	Baoding/2020/BC	06w 35d	CLB83								0	
b23	Baoding/2020/BC	04w27d	CLB94								0	
b24	Baoding/2020/BC	05w31d	CLB99								0	
b25	Hengshui/2020/BC	05w26d	CLB103								0	
b26	Baoding/2020/BC	05w37d	CLB119								0	

b27	Baoding/2020/BC	04w28d	CLB123-1								0	
	**The information of Pullet/layer hens (2019-2020)**											
C1	Baoding/2019/hen	05w30d	CLB62	+							1	St
C2	Baoding/ 2020/hen	19w130d	CLB122	+							1	DP
C3	Baoding/ 2020/hen	19w130d	CLB114	+							1	DP
C4	Baoding/2020/hen	02w11d	CLB86		+	+					2	DP
C5	Baoding/2020/hen	07w45d	CLB24	+	+						2	DP
C6	Baoding/2020/hen	19w130d	CLB118	+							1	DP
C7	Baoding/2020/hen	13w90d	CLB121	+							1	DP St
C8	Baoding/2020/hen	07w45d	CLB25	+							1	DP
C9	Baoding/2020/hen	19w130d	CLB126	+	+		+				3	DP
C10	Baoding/2019/hen	26w180d	C6	+							1	NR
C11	Baoding/2019/hen	07w45d	CLB33	+	+						2	DP
C12	Baoding/2019/hen	19w130d	CLB13			+					1	DP
C13	Baoding/2020/hen	08w70d	CLB22	+	+						2	DP
C14	Baoding/2020/hen	05w35d	CLB59	+							1	St
C15	Baoding/2019/hen	06w38d	CLB91	+							1	RP
C16	Baoding/2020/hen	10w64d	CLB78	+							1	DP RP
C17	Baoding/2019/hen	19w130d	CLB27	+	+	+					3	St
C18	Baoding/2020/hen	04w22d	CLB100	+							1	DP RP
C19	Baoding/2020/hen	10w67d	CLB134	+	+	+		+			4	St
C20	Baoding/2020/hen	10w67d	CLB52	+		+					2	DP
C21	Baoding/2019/hen	08w54d	CLB90			+					1	DP
C22	Baoding/2020/hen	19w130d	CLB56	+		+					2	NR
C23	Baoding/2020/hen	19w130d	CLB11		+						1	DP
C24	Baoding/2020/hen	18w120d	CLB131	+							1	DP
C25	Baoding/2020/hen	10w70d	CLB98	+		+					2	RP
C26	Baoding/2019/hen	20w140d	CLB19	+							1	NR
C27	Baoding/2019/hen	02w12d	CLB110	+							1	DP
C28	Baoding/2020/hen	04w22d	CLB101	+	+						2	DP
C29	Baoding/2020/hen	06w37d	CLB137		+						1	RP
C30	Baoding/2020/hen	04w25d	CLB80	+	+	+					3	DP
C31	Baoding/2020/hen	04w25d	CLB81	+							1	DP
C32	Baoding/2019/hen	05w35d	CLB31	+	+						2	NR
C33	Cangzhou/?/hen	18w120d	CLB67				+				1	St
C34	Cangzhou/2019/hen	08w50d	C2		+						1	St
C35	Cangzhou/2019/hen	20w140d	CLB43	+							1	NR
C36	Cangzhou/2020/hen	08w50d	CLB97	+							1	St
C37	Cangzhou/2020/hen	29w197d	CLB74			+					1	DP
C38	Cangzhou/?/hen	18w120d	CLB72	+							1	DP
C39	Cangzhou/?/hen	20w140d	CLB73	+							1	NR

C40	Cangzhou/2020/hen	28w190d	CLB20	+				1	DP
C41	Cangzhou/2019/hen	05w35d	CLB69		+	+		2	DP
C42	Cangzhou/2019/hen	05w30d	CLB26	+	+	+		3	DP
C43	Cangzhou/2020/hen	29w200d	CLB92				+	1	DP
C44	Cangzhou/2020/hen	18w120d	CLB5	+				1	DP
C45	Cangzhou/2020/hen	20w138d	CLB1		+	+		2	DP
C46	Cangzhou/2019/hen	29w200d	CLB106		+			1	RP
C47	Cangzhou/2019/hen	09w80d	CLB65	+				1	DP
C48	Cangzhou/2019/hen	16w110d	CLB16	+				1	DP
C49	Cangzhou/2020/hen	05w35d	CLB124	+				1	DP
C50	Cangzhou/2020/hen	10w70d	CLB117		+	+		2	DP
C51	Cangzhou/2020/hen	09w75d	CLB53	+				1	DP
C52	Cangzhou/2020/hen	16w108d	CLB112	+				1	DP
C53	Cangzhou/2020/hen	10w64d	CLB93-2		+			1	DP St
C54	Cangzhou/2019/hen	06w39d	CLB50			+		1	DP
C55	Cangzhou/2020/hen	05w35d	CLB125		+			1	DP
C56	Cangzhou/2020/hen	19w140d	CLB14	+				1	DP
C57	Cangzhou/2020/hen	05w35d	CLB127		+			1	DP
C58	Cangzhou/2020/hen	05w35d	CLB128		+			1	DP
C59	Cangzhou/2020/hen	13w85d	CLB109	+		+		2	DP
C60	Hengshui/2019/hen	08w70d	CLB105	+				1	DP
C61	Hengshui/2020/hen	22w150d	CLB7	+				1	NR
C62	Hengshui/2020/hen	35w240d	CLB104	+				1	RP
C63	Hengshui/2019/hen	19w130d	CLB58		+			1	DP
C64	Hengshui/2020/hen	26w180d	CLB75	+				1	DP
C65	Hengshui/2020/hen	09w60d	CLB60	+				1	NR
C66	Langfang/2020/hen	14w95d	CLB41	+		+		2	DP
C67	Shijiazhuang/2019/hen	08w50d	CLB95	+				1	St
C68	Shijiazhuang/2020/hen	19w130d	CLB84	+		+		2	DP
C69	Shijiazhuang/2019/hen	05w30d	CLB47	+				1	St
C70	Shijiazhuang/2020/hen	26w180d	CLB133	+	+			2	NR
C71	Shijiazhuang/2020/hen	03w18d	CLB61	+				1	DP
C72	Shijiazhuang/2019/hen	09w60d	CLB42	+				1	DP
C73	Shijiazhuang/2019/hen	23w160d	CLB68	+				1	DP
C74	Xingtai/2020/hen	18w120d	CLB2	+				1	St
C75	Baoding/2019/hen	02w10d	CLB54					0	
C76	Baoding/2019/hen	02w13d	CLB49					0	
C77	Cangzhou/2019/hen	05w35d	C4					0	
C78	Cangzhou/2020/hen	06w37d	CLB132					0	
C79	Hengshui/2020/hen	07w45d	CLB48					0	
C80	Shijiazhuang/2020/hen	07w46d	CLB12					0	

C81	Baoding/2019/hen	08w50d	CLB79	0
C82	Baoding/2019/hen	09w60d	CLB108	0
C83	Baoding/2019/hen	09w60d	CLB108-1	0
C84	Baoding/2019/hen	09w60d	CLB30	0
C85	Baoding/2019/hen	09w60d	CLB87	0
C86	Cangzhou/2020/hen	10w64d	CLB93	0
C87	Cangzhou/2020/hen	10w64d	CLB93-1	0
C88	Cangzhou/2020/hen	10w64d	CLB93-3	0
C89	Cangzhou/2020/hen	10w64d	CLB93-4	0
C90	Cangzhou/2020/hen	10w64d	CLB93-5	0
C91	Hengshui/2020/hen	10w65d	CLB10	0
C92	Cangzhou/2019/hen	10w70d	CLB89	0
C93	Baoding/2019/hen	10w70d	C1	0
C94	Baoding/2019/hen	12w80d	CLB38	0
C95	Baoding/2020/hen	18w120d	CLB3	0
C96	Shijiazhuang/2020/hen	18w120d	CLB102	0
C97	Cangzhou/2020/hen	18w120d	CLB107	0
C98	Cangzhou/2020/hen	18w120d	CLB130	0
C99	Shijiazhuang/2020/hen	18w120d	CLB55	0
C100	Baoding/2020/hen	19w130d	CLB63	0
C101	Baoding/2020/hen	19w130d	CLB115	0
C102	Baoding/2020/hen	19w130d	CLB116	0
C103	Baoding/2020/hen	19w130d	CLB135	0
C104	Baoding/2019/hen	22w150d	CLB9	0
C105	Shijiazhuang/2020/hen	22w150d	CLB21	0
C106	Cangzhou/2019/hen	23w160d	CLB44	0
C107	Cangzhou/2020/hen	23w160d	CLB4	0
C108	Hengshui/2019/hen	25w170d	C5	0
C109	Baoding/2019/hen	25w170d	CLB39	0
C110	Baoding/2019/hen	25w170d	CLB40	0
C111	Baoding/2020/hen	26w180d	CLB45	0
C112	Baoding/2020/hen	26w180d	CLB46	0
C113	Cangzhou/2019/hen	28w190d	CLB34	0
C114	Baoding/2020/hen	28w190d	CLB113	0
C115	Hengshui/2020/hen	29w200d	CLB23	0
C116	Cangzhou/2019/hen	29w200d	CLB28	0
C117	Hengshui/2019/hen	29w200d	CLB35	0
C118	Cangzhou/2019/hen	29w200d	CLB66	0
C119	Cangzhou/2020/hen	29w200d	CLB76	0
C120	Cangzhou/2020/hen	29w200d	CLB85	0
C121	Shijiazhuang/2020/hen	35w240d	CLB8	0

C122	Cangzhou/2020/hen	36w250d	CLB36								0	
C123	Cangzhou/2020/hen	42w290d	CLB71								0	
C124	Baoding/2020/hen	49w340d	CLB138								0	
	**The information of Pullet/layer hens (2021)**											
L1	Baoding/2021/hen	06w42d	Ly05	+		+					2	NR
L2	Baoding/2021/hen	05w35d	Ly06	+		+					2	NR
L3	Shijiazhuang/2021/hen	06w38d	Ly20		+	+					2	NR
L4	Cangzhou/2021/hen	04w25d	Ly26		+	+					2	NR
L5	Shijiazhuang/2021/hen	08w50d	Ly29		+	+					2	NR
L6	Baoding/2021/hen	19w130d	Ly44	+			+				2	NR
L7	Baoding/2021/hen	13w87d	Ly01	+							1	NR
L8	Cangzhou/2021/hen	08w50d	Ly03			+					1	NR
L9	Baoding/2021/hen	22w150d	Ly04			+					1	NR
L10	Baoding/2021/hen	13w90d	Ly09	+							1	NR
L11	Baoding/2021/hen	22w150d	Ly11	+							1	NR
L12	Baoding/2021/hen	13w87d	Ly12	+							1	NR
L13	Baoding/2021/hen	13w90d	Ly13			+					1	NR
L14	Cangzhou/2021/hen	15w105d	Ly14			+					1	NR
L15	Langfang/2021/hen	17w115d	Ly15			+					1	NR
L16	Cangzhou/2021/hen	23w160d	Ly18			+					1	NR
L17	Baoding/2021/hen	08w52d	Ly2?		+						1	NR
L18	Baoding/2021/hen	06w42d	Ly22		+						1	NR
L19	Shijiazhuang/2021/hen	08w50d	Ly23		+						1	NR
L20	Baoding/2021/hen	05w35d	Ly25	+							1	NR
L21	Baoding/2021/hen	09w58d	Ly27			+					1	NR
L22	Baoding/2021/hen	10w65d	Ly28			+					1	NR
L23	Cangzhou/2021/hen	06w40d	Ly30	+							1	NR
L24	Shijiazhuang/2021/hen	09w60d	Ly31		+						1	NR
L25	Hengshui/2021/hen	09w60d	Ly32	+							1	NR
L26	Baoding/2021/hen	10w64d	Ly33	+							1	NR
L27	Hengshui/2021/hen	13w90d	Ly34	+							1	NR
L28	Cangzhou/2021/hen	14w95d	Ly35			+					1	NR
L29	Cangzhou/2021/hen	05w30d	Ly36			+					1	NR
L30	Baoding/2021/hen	16w110d	Ly37	+							1	NR
L31	Cangzhou/2021/hen	19w128d	Ly38	+							1	NR
L32	Cangzhou/2021/hen	19w130d	Ly39	+							1	NR
L33	Cangzhou/2021/hen	22w150d	Ly40	+							1	NR
L34	Baoding/2021/hen	20w140d	Ly41	+							1	NR
L35	Cangzhou/2021/hen	20w140d	Ly42	+							1	NR
L36	Baoding/2021/hen	19w130d	Ly45	+							1	NR
L37	Baoding/2021/hen	19w130d	Ly46	+							1	NR

L38	Baoding/2021/hen	26w160d	Ly47	+			1	NR
L39	Cangzhou/2021/hen	26w160d	Ly48	+			1	NR
L40	Cangzhou/2021/hen	03w20d	Ly49			+	1	NR
L41	Hengshui/2021/hen	10w70d	Ly50			+	1	NR
L42	Cangzhou/2021/hen	05w30d	Ly54		+		1	NR
L43	Hengshui/2021/hen	06w40d	Ly55			+	1	NR
L44	Baoding/2021/hen	05w31d	Ly56		+		1	NR
L45	Cangzhou/2021/hen	08w55d	Ly57		+		1	NR
L46	Cangzhou/2021/hen	13w90d	Ly58	+			1	NR
L47	Cangzhou/2021/hen	20w138d	Ly59	+			1	NR
L48	Cangzhou/2021/hen	05w35d	Ly61	+			1	NR
L49	Cangzhou/2021/hen	05w32d	Ly62	+			1	NR
L50	Baoding/2021/hen	09w57d	Ly63	+			1	NR
L51	Cangzhou/2021/hen	14w92d	Ly02				0	NR
L52	Cangzhou/2021/hen	05w31d	Ly43				0	NR
L53	Baoding/2021/hen	16w110d	Ly29				0	NR
L54	Cangzhou/2021/hen	19w128d	Ly38				0	NR
L55	Cangzhou/2021/hen	19w130d	Ly16				0	NR
L56	Cangzhou/2021/hen	22w150d	Ly70				0	NR
L57	Baoding/2021/hen	20w140d	Ly16-1				0	NR
L58	Cangzhou/2021/hen	20w140d	Ly71				0	NR
L59	Baoding/2021/hen	19w130d	Ly65				0	NR
L60	Baoding/2021/hen	19w130d	Ly66				0	NR
L61	Baoding/2021/hen	26w160d	Ly43				0	NR

Abbreviations: ANV, avian nephritis virus; CAstV, chicken astrovirus; ARe, avian reovirus; ARoV, avian rotavirus; IBV, infectious bronchitis virus; FAdV-I, fowl adenovirus of group-I; ChPV, chicken parvovirus.RP, Respiratory problems; DP, Digestive problems; St, Stunting; NR, Not reported.

Supplementary Table S2. Parvovirus (PV) reference strain information.

Strain	Country	Organism	Host	GenBank
GX-CH-PV-15	CN/2015	Chicken parvovirus	chicken	KX133422.1
GX-CH-PV-10	CN/2015	Chicken parvovirus	chicken	KX133417.1
GX-CH-PV-11	CN/2015	Chicken parvovirus	chicken	KX133418.1
GX-CH-PV-8	CN/2015	Chicken parvovirus	chicken	KX133415.1
GX-CH-PV-5	CN/2014	Chicken parvovirus	chicken	KX133426.1
CH-CH-PV-4	CN/2014	Chicken parvovirus	chicken	KX084401.1
CH-CH-PV-2	CN/2014	Chicken parvovirus	chicken	KX084400.1
GA/1478/2003	US/2003	Chicken parvovirus	chicken	MN782010.1
GA/1477/2005	US/2005	Chicken parvovirus	chicken	MN782009.1
GA/1472/2004	US/2004	Chicken parvovirus	chicken	MN782008.1
ABU-P1	HU/2009	Chicken parvovirus	Chicken	NC_024452.1
GX-CH-PV-27	CN/2017	Chicken parvovirus	Gallus gallus	MG602517.1
GX-CH-PV-24	CN/2017	Chicken parvovirus	Gallus gallus	MG602514.1
GX-CH-PV-23	CN/2016	Chicken parvovirus	Gallus gallus	MG602513.1

GX-CH-PV-22	CN/2016	Chicken parvovirus	Gallus gallus	MG602512.1
ADL120035	KR/2012	Chicken parvovirus	Gallus gallus	KJ486490.1
ADL120686	KR/2012	Chicken parvovirus	Gallus gallus	KJ486491.1
ADL120019	KR/2012	Chicken parvovirus	Gallus gallus	KJ486489.1
IPV	BR/2012	Chicken parvovirus	Gallus gallus	KU569162.1
TuPV JO11	US/2014	Turkey parvovirus	Meleagris gallopavo	KM598421.1
TuPV 1090	US/2014	Turkey parvovirus	Meleagris gallopavo	KM598420.1
TuPV 1030	US/2014	Turkey parvovirus	Meleagris gallopavo	KM598418.1
GX-Tu-PV-3	CN/2015	Turkey parvovirus	Turkey	KX084398.1
GX-Tu-PV-2	CN/2014	Turkey parvovirus	Turkey	KX084397.1
GX-Tu-PV-1	CN/2015	Turkey parvovirus	Turkey	KX084396.1
1078	US/2009	Turkey parvovirus	Turkey	NC_024454.1
260	US/2009	Turkey parvovirus	Turkey	NC_038534.1
DS15	CN/2015	Duck parvovirus	cherry valley duck	KX384726.1

Abbreviations: CN, China; UK, United Kingdom; IN, India; US, United States; PL, Poland; SF, South Africa; NL, Netherlands; JP , Japan; AUS, Australia; BR, Brazil.

Supplementary Table S3. Avian nephritis virus (ANV) and chicken astrovirus (CAstV) reference strain information.

Viruses	Strain	Country	Organism	Host	GenBank
ANV	-	JP/2000	ANV-2	Chicken	AB046864.1
ANV	ANV-VF08-60	UK/2008	ANV-2	Chicken	HQ330505.1
ANV	VF16-03-164b	UK/2016	ANV-3	Chicken	MT585644.1
ANV	Avian AstV Brazil 57-3	BR/2014	ANV-4	Chicken	KU711072.1
ANV	ANV-VF07-13/7	UK/2007	ANV-4	Chicken	HQ330482.1
ANV	ANV/CHN/BJCP510-2/2018	CN/2018	ANV-5	Gallus gallus	MN732558.1
ANV	Avian AstV Brazil 48-5	BR/2014	ANV-6	Chicken	KU711068.1
ANV	Avian AstV Brazil 48-9	BR/2014	ANV-6	Chicken	KU711069.1
ANV	NSW-3a	AUS/2009	ANV-7	Chicken	KM985692.1
CAstV	P22-18.8.00	NL/2004	Ai	Gallus gallus	JN582318.1
CAstV	612	SF/1994	Ai	Gallus gallus	JN582317.1
CAstV	VF08-56	UK/2008	Ai	Gallus gallus	JN582319.1
CAstV	CAstV/Poland/G059/2014	PL/2014	Aiii	Gallus gallus	KT886453.1
CAstV	1010	UK/2004	Bi	Gallus gallus	JN582306.1
CAstV	AAstV/Chicken/CHN/2018/CZ1801	CN/2018	Bi	Gallus gallus	MN807051.1
CAstV	CAstV/CHN/GDYHTJ718-6/2018	CN/2018	Bi	Chicken	MN725026.1
CAstV	4175	US/2006	Bii	Gallus gallus	JF832365.1
CAstV	CAstV/INDIA/ANAND/2016	IN/2016	Biii	Chicken	KY038163.1
CAstV	PDRC/200/East Zone	IN /2011	Biii	Gallus gallus	JX945853.1
CAstV	CAstV/CA-AB/Chicken/17-0773b/17	CA/2017	Biv	Chicken	MT789783.1
CAstV	CAstV/CA-SK/Chicken/19-0935/19	CA/2019	Biv	Chicken	MT789786.1
CAstV	CkP5	US/2016	Biv	Chicken	KX397576.1

Abbreviations: JP , Japan; UK, United Kingdom; CN, China; BR, Brazil; AUS, Australia; NL, Netherlands; PL, Poland; SF, South Africa; US, United States; IN, India; CA, Canada.

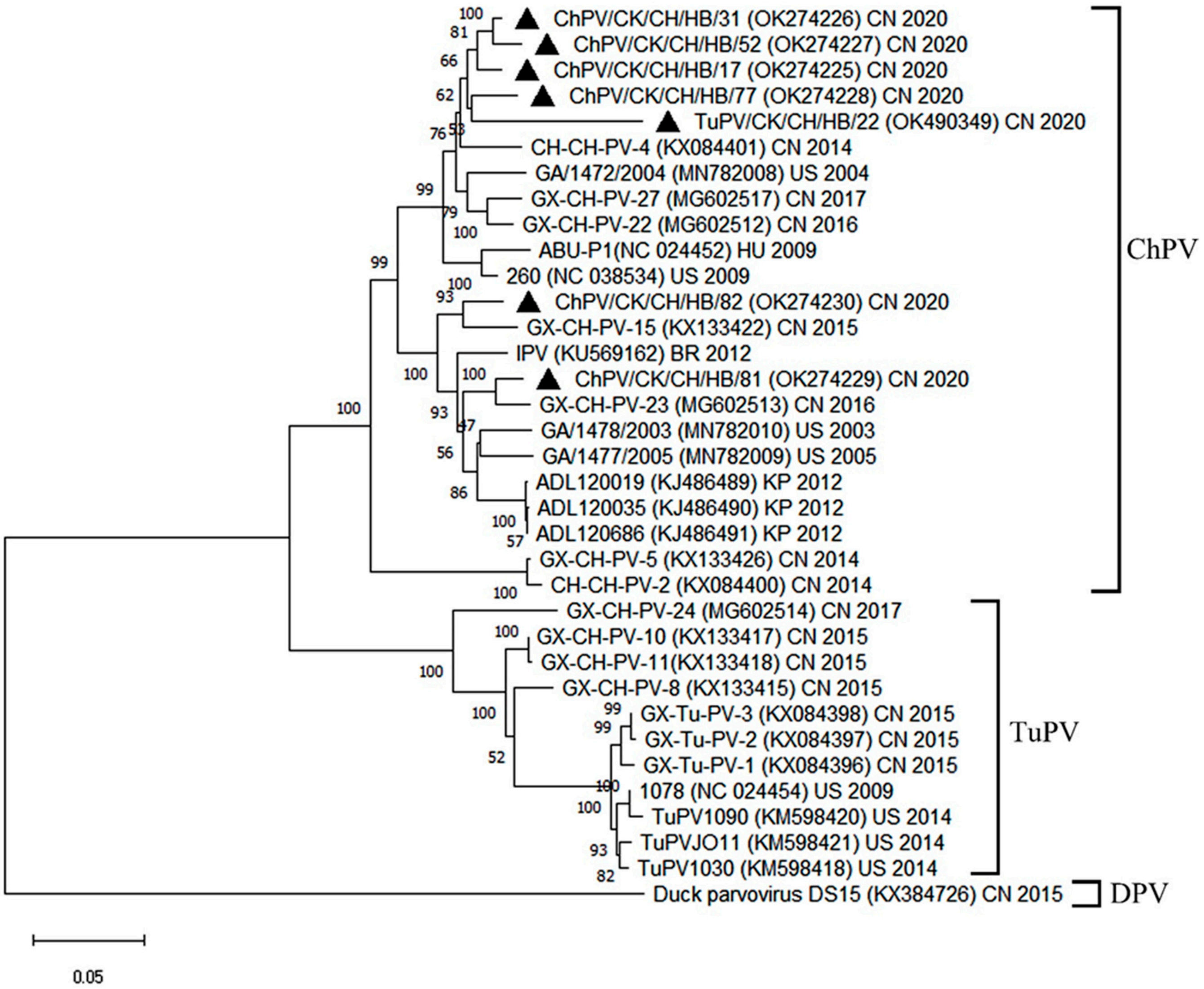
ChPV/CK/CH/HB/31 (OK274226) CN 2020
ChPV/CK/CH/HB/52 (OK274227) CN 2020
ChPV/CK/CH/HB/17 (OK274225) CN 2020
ChPV/CK/CH/HB/77 (OK274228) CN 2020
TuPV/CK/CH/HB/22 (OK490349) CN 2020
CH-CH-PV-4 (KX084401) CN 2014
GA/1472/2004 (MN782008) US 2004
GX-CH-PV-27 (MG602517) CN 2017
GX-CH-PV-22 (MG602512) CN 2016
ABU-P1(NC 024452) HU 2009
260 (NC 038534) US 2009
ChPV/CK/CH/HB/82 (OK274230) CN 2020
GX-CH-PV-15 (KX133422) CN 2015
IPV (KU569162) BR 2012
ChPV/CK/CH/HB/81 (OK274229) CN 2020
GX-CH-PV-23 (MG602513) CN 2016
GA/1478/2003 (MN782010) US 2003
GA/1477/2005 (MN782009) US 2005
ADL120019 (KJ486489) KP 2012
ADL120035 (KJ486490) KP 2012
ADL120686 (KJ486491) KP 2012
GX-CH-PV-5 (KX133426) CN 2014
CH-CH-PV-2 (KX084400) CN 2014
GX-CH-PV-24 (MG602514) CN 2017
GX-CH-PV-10 (KX133417) CN 2015
GX-CH-PV-11(KX133418) CN 2015
GX-CH-PV-8 (KX133415) CN 2015
GX-Tu-PV-3 (KX084398) CN 2015
GX-Tu-PV-2 (KX084397) CN 2015
GX-Tu-PV-1 (KX084396) CN 2015
1078 (NC 024454) US 2009
TuPV1090 (KM598420) US 2014
TuPVJO11 (KM598421) US 2014
TuPV1030 (KM598418) US 2014
Duck parvovirus DS15 (KX384726) CN 2015
ChPV
TuPV
DPV
0.05

(**A**)

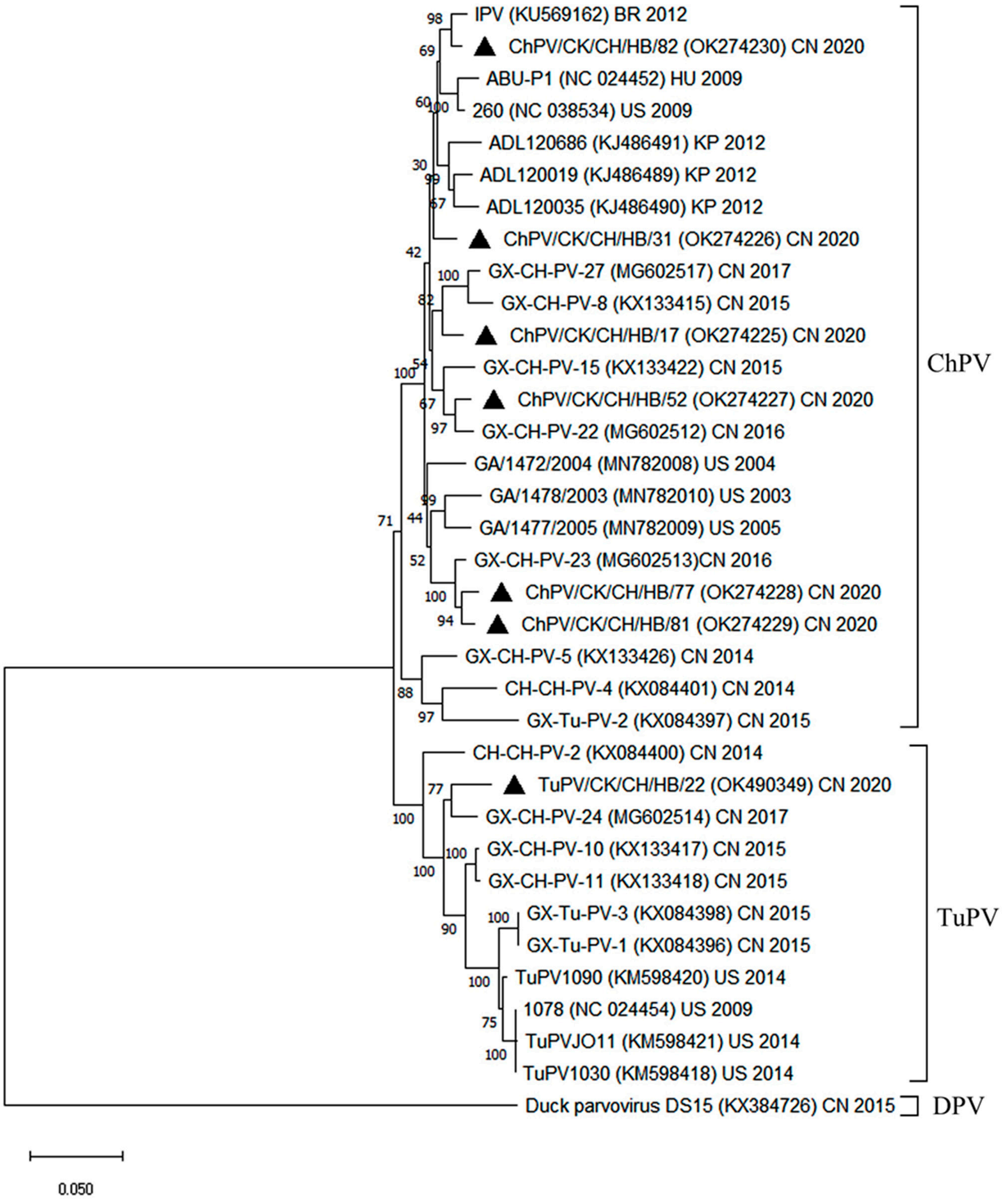

(B)

Supplementary Figure S1. Phylogenetic tree based on the full coding sequence of the VP1 (A) and NS (B) genes of parvovirus. Molecular Evolutionary Genetics Analysis version 11 were used for phylogenetic tree reconstruction using the neighbor-joining algorithm with 1000 bootstrap replicates. The tree was drawn to scale, with branch lengths in the same units as those of the evolutionary distances used to infer the phylogenetic tree. Accession numbers are shown in parentheses. Strains with ▲ represent

PV sequenced in this paper. Sequence of duck parvovirus was used as the out-group control. CN, China; US, United States; HU, Hungary; BR, Brazil; KR, South Korea.
